# An Antiretroviral/Zinc Combination Gel Provides 24 Hours of Complete Protection against Vaginal SHIV Infection in Macaques

**DOI:** 10.1371/journal.pone.0015835

**Published:** 2011-01-05

**Authors:** Jessica Kenney, Meropi Aravantinou, Rachel Singer, Mayla Hsu, Aixa Rodriguez, Larisa Kizima, Ciby J. Abraham, Radhika Menon, Samantha Seidor, Anne Chudolij, Agegnehu Gettie, James Blanchard, Jeffrey D. Lifson, Michael Piatak, Jose A. Fernández-Romero, Thomas M. Zydowsky, Melissa Robbiani

**Affiliations:** 1 Population Council, New York, New York, United States of America; 2 Aaron Diamond AIDS Research Center, Rockefeller University, New York, New York, United States of America; 3 Tulane National Primate Research Center, Tulane University, Covington, Louisiana, United States of America; 4 AIDS and Cancer Virus Program, SAIC-Frederick, National Cancer Institute, Frederick, Maryland, United States of America; University of Pittsburgh, United States of America

## Abstract

**Background:**

Repeated use, coitus-independent microbicide gels that do not contain antiretroviral agents also used as first line HIV therapy are urgently needed to curb HIV spread. Current formulations require high doses (millimolar range) of antiretroviral drugs and typically only provide short-term protection in macaques. We used the macaque model to test the efficacy of a novel combination microbicide gel containing zinc acetate and micromolar doses of the novel non-nucleoside reverse transcriptase inhibitor MIV-150 for up to 24 h after repeated gel application.

**Methods and Findings:**

Rhesus macaques were vaginally challenged with SHIV-RT up to 24 h after repeated administration of microbicide versus placebo gels. Infection status was determined by measuring virologic and immunologic parameters. Combination microbicide gels containing 14 mM zinc acetate dihydrate and 50 µM MIV-150 afforded full protection (21 of 21 animals) for up to 24 h after 2 weeks of daily application. Partial protection was achieved with the MIV-150 gel (56% of control at 8 h after last application, 11% at 24 h), while the zinc acetate gel afforded more pronounced protection (67% at 8–24 h). Marked protection persisted when the zinc acetate or MIV-150/zinc acetate gels were applied every other day for 4 weeks prior to challenge 24 h after the last gel was administered (11 of 14 protected). More MIV-150 was associated with cervical tissue 8 h after daily dosing of MIV-150/zinc acetate versus MIV-150, while comparable MIV-150 levels were associated with vaginal tissues and at 24 h.

**Conclusions:**

A combination MIV-150/zinc acetate gel and a zinc acetate gel provide significant protection against SHIV-RT infection for up to 24 h. This represents a novel advancement, identifying microbicides that do not contain anti-viral agents used to treat HIV infection and which can be used repeatedly and independently of coitus, and underscores the need for future clinical testing of their safety and ability to prevent HIV transmission in humans.

## Introduction

There is a critical need for safe and effective microbicides that women worldwide can use repeatedly and independently from the time of coitus, to prevent sexual transmission of human immunodeficiency virus (HIV) and other sexually transmitted infections (STIs). While promising, candidate microbicide gels containing agents that act on specific viral targets and/or antagonists of cell-virus interactions typically require mM or mg/ml amounts of the drug to significantly protect against mucosal infection with immunodeficiency virus in macaques [1], [2], [3], [4], [5], [6], [7]. Notably, the 1% (1 mg/ml) tenofovir gel that protected macaques against repeated vaginal infection when given 30 min prior to each challenge [5], was shown to be effective in reducing HIV acquisition in women by 39% when applied at least 12 h before and no more than 12 h after intercourse in the CAPRISA 004 trial [8]. This provides the first proof of concept that topical microbicides can limit HIV spread in humans and that this was predicted from the macaque studies.

In microbicides it is preferable to avoid first line anti-HIV agents that are used to treat HIV-infected people, or agents with the potential to induce class- or cross-resistance to them [9], [10]. There was no evidence of tenofovir resistance in those individuals who became infected in the CAPRISA 004 trial [8]. However, tenofovir is used to treat HIV infection [11], therefore increasing the chances of the transmission of tenofovir resistant viruses in the future. An additional attribute that will increase the success of a microbicide formulation is the ability to exhibit long lasting protection that would allow gels to be used independently of intercourse and thus be useful in real-world settings. Therefore, identifying a formulation that (i) contains agents that are active against viruses already resistant to drugs in clinical use and (ii) provides protection when applied independent of intercourse is vital.

We explored the use of MIV-150, a novel non-nucleoside reverse transcriptase inhibitor (NNRTI) that is not used in current HIV therapies, combined with zinc acetate and formulated in carrageenan. NNRTIs are non-competitive inhibitors of RT. Carrageenan alone (Carraguard®; 95% lambda and 5% kappa carrageenan) was not effective at preventing HIV infection in women [12], but the intrinsic rheological properties [13], stability, acceptability, and safety of a carrageenan-based gel [12], [14], [15], [16], [17], [18], [19] make it a useful vehicle to deliver anti-HIV drugs. MIV-150 is a novel, potent NNRTI, which binds tightly to the HIV reverse transcriptase (RT), and has strong antiviral (IC_50_ of <1 nM) and potentially virucidal (IC_50_ of 400 nM) activity against R5 and X4 viruses [13], [20], [21]. Moreover, MIV-150 possesses a favorable resistance profile: it is effective against HIV-1 harboring common single mutations in the RT gene, requires two-to-three mutations (L100I, K103N, Y181C) to increase the IC_50_>10-fold, and takes about twice as long to select HIV-1 resistance *in vitro* compared to other NNRTIs like Nevirapine and Efavirenz (Fernández-Romero unpublished). Notably, the L100I/K103N double mutant resistant to Efavirenz had reduced susceptibility to MIV-150. The IC_50_ of MIV-150 against this double mutant (0.9 µM vs 0.7 nM against wild type) remained at least 10-fold less than that of Efavirenz (>10 µM vs 0.6 nM against wild type). However, the Y181C mutant resistant to Nevirapine remained fully sensitive to MIV-150 (0.2 nM vs 0.7 nM against wild type). Importantly, MIV-150 also possesses a potent memory effect, since cells exposed to MIV-150 *in vitro* remain resistant to infection for up to 5 d (Fernández-Romero unpublished).

Formulating MIV-150 in a carrageenan gel has yielded encouraging results to date. *In vitro* studies demonstrated the additive effects of MIV-150 and carrageenan [13]. MIV-150 also prevented dendritic cell (DC)-facilitated infection of CD4^+^ T cells at nM concentrations [20]. In addition, *in vivo* efficacy of 500 µM MIV-150 in carrageenan was confirmed against our chimeric SHIV-RT (SIVmac239 with HIV-1 RT) when applied topically 30 min prior to vaginal challenge of either naïve or HSV-2 infected macaques, despite the increased susceptibility to SHIV-RT infection of the latter [20], [22].

*In vitro* studies suggest that zinc salts have activity against HIV as well as other viruses, including HSV-2 [23], [24], [25], infection with which has been shown to facilitate HIV transmission [26], [27], [28]. However, documentation of anti-viral activity *in vitro* is complicated by the toxicity of zinc in many cell-based assays, underscoring the need to conduct *in vivo* studies of zinc's anti-viral activity. Moreover, we found that zinc acetate delivered in carrageenan (but not zinc acetate in solution or in the hydroxyethyl cellulose placebo) protects mice against high dose vaginal challenge with HSV-2 (Fernández-Romero, unpublished). Thus, gels containing zinc acetate represent a promising approach to impede HIV transmission both directly by virtue of anti-HIV activity, and indirectly by reducing other STIs. In addition, since condom use might be reduced upon introduction of a microbicide [29], a formulation that targets HIV as well as other STIs, like HSV-2, is especially desirable.

To assess the potential of a novel antiretroviral/zinc combination microbicide in a manner designed to simulate how women will use gels, we evaluated the anti-viral efficacy of repeated vaginal application of MIV-150 and zinc acetate in a macaque vaginal challenge model. Herein we show that daily usage of gels containing low dose MIV-150 (50 µM) afforded >50% protection from infection for up to 8 h after 2 weeks of daily application, and that zinc acetate alone protected >65% of macaques from infection for up to 24 h. Notably, the combination of low dose MIV-150 and zinc acetate provided complete protection from infection for at least 24 h. Both zinc acetate alone and the MIV-150/zinc acetate combination gels still markedly reduced infection when applied every other day. These data will advance the development of coitally-independent combination microbicide gels to limit HIV spread.

## Methods

### Ethics Statement

Adult female Chinese rhesus macaques (*Macaca mulatta*) were housed and cared for in compliance with the regulations under the Animal Welfare Act, the Guide for the Care and Use of Laboratory Animals, at Tulane National Primate Research Center (TNPRC; Covington, LA). Animals were monitored continuously by veterinarians to ensure their welfare. Veterinarians at the TNPRC Division of Veterinary Medicine have established procedures to minimize pain and distress through several means. Monkeys were anesthetized with ketamine-HCl (10 mg/kg) or tiletamine/zolazepam (6 mg/kg) prior to all procedures. Preemptive and post procedural analgesia (buprenorphine 0.01 mg/kg) was required for procedures that would likely cause more than momentary pain or distress in humans undergoing the same procedures. The above listed anesthetics and analgesics were used to minimize pain or distress associated with this study in accordance with the recommendations of the Weatherall Report. Any sick animals were euthanized using methods consistent with recommendations of the American Veterinary Medical Association (AVMA) Panel on Euthanasia. All studies were approved by the Animal Care and Use Committee of the TNPRC (OLAW assurance #A4499-01) and in compliance with animal care procedures. TNPRC is accredited by the Association for Assessment and Accreditation of Laboratory Animal Care (AAALAC#000594).

### Animal treatments and challenge

Animals tested negative for simian type D retroviruses, simian T cell leukemia virus-1, and SIV prior to use in the efficacy studies. The animals were sexually mature (ranging from 4–12 years old) and their weights ranged from 4–10 kg. There was no evidence of a correlation between an animal's age at the time of challenge and infection status. Uninfected and healthy SHIV-RT infected animals available from completed microbicide studies were used for the PK and biomarker studies. All blood (no more than 10 ml/kg/month), fluids, and superficial lymph node biopsies were transported overnight from the TNPRC to our laboratories in New York by overnight courier service for processing and analysis.

Five weeks prior to virus challenge, animals received a single 30 mg i.m. injection of Depo-Provera to thin the vaginal epithelium as well to control cycling [30]. At the designated times, single 3 ml applications or repeated 2 ml applications of microbicide candidate gel (versus the vehicle carrageenan and methyl cellulose [MC] controls) were applied atraumatically into the vaginal vault with a pliable pediatric feeding tube either before or after challenge with 0.5 ml (for repeated gel treated animals) or 1 ml (for animals receiving one gel dose) of 10^3^ TCID_50_ SHIV-RT (SIVmac239 and HIV-1_hxb2_ RT). A supine position was maintained for all animals, to allow absorption of virus for 20 min post challenge. There was no evidence of leakage from the animals. Control gel-treated animals were included with every challenge. The infection frequencies for animals treated at different times with the MC placebo were comparable and so those data have been pooled to provide the MC dataset. This was also the case for animals treated with carrageenan. Individual animal information is summarized in Tables S1–S3. For the PK/biomarker studies the animals were treated with Depo-Provera prior to gel treatment just as in the efficacy studies, but without the virus challenge.

### Virus stock

The original SHIV-RT stocks were grown in PHA-activated human peripheral blood mononuclear cells (PBMCs) (kindly provided by Disa Böttiger, Medivir AB, Sweden). A subsequent stock was generated from this in PHA-activated macaque PBMCs. Stocks were re-titered using the 174xCEM cell line (NIH AIDS Research & Reference Reagent Program), and TCID_50_ values were calculated using the Reed and Muench formula. Both stocks infect with comparable frequency *in vivo*.

### Microbicide formulations

The 2.5% (w/w) MC placebo gels (Lot numbers 32805, 110205, 032807, 011008, 080602A2005SR, 080804A2005MR, 090217A2005MR, 090610A2005MR) (Fisher) and the 3% (w/w) carrageenan vehicle PC-515 (Lot numbers 32805, 010908, 080805A515SR, 090127A515SR, 090612A515MR) were used as controls [31]. MC was originally used to parallel the human studies testing Carraguard where MC was the placebo [12] and to rule out any barrier effect of the carrageenan vehicle as was observed in our earlier macaque studies [20]. PC-817 (Lot numbers 32805, 011508, 032707-A, 080603A817MR, 080811A817MR, and 090210A817MR) and PC-815 (Lot numbers 080807A815SR, 090209A815MR, 090609A815MR) contained 3% (w/w) carrageenan, either 500 µM (PC-817) or 50 µM (PC-815) MIV-150, and 1% DMSO (Sigma, St. Louis, MO). PC-1005 (Lot numbers 080604A1005MR, 080810A1005SR, 090205A1005MR, 100126A1005MR) contained 3% (w/w) carrageenan, 50 µM MIV-150, 14 mM zinc acetate dihydrate, and 1% DMSO. PC-707 (Lot numbers 090202A707MR, 090204A707MR, 100127A707MR) contained 3% (w/w) carrageenan and 14 mM zinc acetate dihydrate. Gels were stored at room temperature and used for the studies within 7–28 days of formulation. Gel viscosity and anti-HIV activity were verified for each Lot prior to *in vivo* use. The pH of the gels was buffered to 6.8±0.2 in the final manufacturing step, so any change in pH due to the addition of zinc acetate was neutralized. MIV-150 was developed by Medivir AB (Sweden) and licensed to the Population Council for development as a microbicide.

### Cell isolation and sample collection

PBMCs were isolated from EDTA blood using Ficoll-Hypaque density gradient centrifugation (Amersham Pharmacia Biotech, Uppsala, Sweden) as already described [32]. RPMI 1640 (Invitrogen/GIBCO, Carlsbad, CA, USA) culture medium containing 2 mM L-glutamine (Invitrogen/GIBCO) 10 mM HEPES (N-2-hydroxyethylpiperazine-N'-2-ethanesulfonic acid) (Invitrogen/GIBCO), 50 µM 2-mercaptoethanol (Sigma) penicillin (100 U/ml) and streptomycin (100 µg/ml) (Invitrogen/GIBCO) and 1% heparinized human plasma (Innovative Research, Southfield, MI) was used for final re-suspension of PBMCs. Medium with 10% HI-FBS (heat inactivated fetal bovine serum) (Invitrogen/GIBCO) instead of 1% human plasma was used for the PBMC-174xCEM co-cultures. DNA was isolated from lymph nodes as described with the DNeasy® Blood & Tissue Kit (Qiagen Sciences, MD USA). DNA was eluted in 50 µl RNAse/DNAse free water (Invitrogen/GIBCO). Vaginal swabs and plasma were collected as previously described [22]. Vaginal pH was determined by inserting pH-indicator strip (EMO Chemical Inc., Gibbstown, NJ) into the vaginal vault for 5 minutes to saturate the pH paper with vaginal fluid.

### Virus detection

Plasma viral RNA copy numbers were determined by quantitative RT-PCR as previously reported [33]. The 6 animals with blips were further tested by quantitative SIV *gag* PCR for viral DNA levels in lymph node biopsies taken 30–57 weeks post challenge [34].

### Immune parameters

SIV-specific IFN-γ T cell responses were monitored by ELISPOT using AT-2 SIV (versus the no virus microvesicle controls) as the stimuli [35] and SIV-specific antibodies by ELISA [36]. Antibody positivity was defined as having positive OD values above background baseline samples at 4–8 weeks post challenge and IFN-γ positivity was defined by at least 50 SIV-specific IFNγ spot forming cells (SFCs) per 10^6^ PBMCs on more than one time point post challenge. Cell-free vaginal fluids were analyzed for chemokine and cytokine expression using the monkey-reactive Beadlyte human 14-plex Detection System according to the manufacturer's instructions (Invitrogen). This assay recognized the macaque IL1-β, CXCL10, IL-6, CCL5, CCL3, GM-SCF, CCL4, CCL2, IFN-γ, TNF-α, IL-3, IL-2, IL-4, CXCL8. The Luminex 200 (Luminex, Austin, TX) and StarStation softwares (Applied Cytometry Systems, Sacramento, CA) were used to analyze samples.

### MIV-150 Radioimmunoassay (RIA)

Plasma and vaginal swabs were treated for 30 min at room temperature with NP40 at a final concentration of 1% to inactivate any infectious agent. The samples were stored at −80°C until the RIA was performed. The RIA for MIV-150 was an indirect extraction based assay adapted from Kumar et al [37]. MIV-150 [38] was synthesized by catalyzed exchange with tritium gas and purified by preparative HPLC (American Radiolabeled Chemicals, Inc., St. Louis, MO) and a rabbit polyclonal Ab against MIV-150 was custom prepared by Pacific Immunology Corp. (Ramona, CA). The assay was optimized and validated for detection of MIV-150 in plasma, vaginal swabs, and cervical/vaginal tissues with a level of sensitivity of 2.7 nM (Rodriguez, unpublished). MIV-150 concentration in the samples was calculated by interpolation with the standard curve using a curve fitting procedure (logistic 4-parameter model).

### Cloning and sequencing SHIV-RT genes

Viral RNA was extracted from 1 ml aliquots of plasma from infected animals, using the Qiagen Viral RNA Isolation Kit (Qiagen, Valencia CA, USA) according to the manufacturer's instructions and eluted in 60 µl RNAse/DNAse-free water (Invitrogen). A PBS control was run in parallel to ensure lack of contaminating RNA. Viral RNA was transcribed into cDNA with the Superscript III Reverse Transcriptase Kit (Invitrogen). RT genes in viral cDNA were amplified by PCR, using Pfu Ultra II Hotstart Polymerase (Agilent Technologies, Santa Clara, CA) and primers RT amp 5′ (5′ upstream of HIV RT, within SIV *pol*, 5-TACTAAAGAATACAAAAATGTAGA-3), and RT amp 3′ (3′ downstream of RT 5-CTCTGTGGATTGTATGGTACCCC-3). Due to the lower RNA viral load, an additional nested PCR reaction was performed for animal IE83 with primers 5-TAAATTTTCCCATTAGCCC-3 and 5-TCTTCTGTTAGTGGTATTA-3. PCR amplifications were carried out using the MyCycler Thermal Cycler (Bio-Rad, Laboratories, Inc., Hercules, CA). After activation of the Taq polymerase at 94°C for 5 min, DNA was amplified for 30 cycles, each at 94°C for 1 min, 44°C for 1 min, 72°C for 2 min, with a final extension at 72°C for 5 min. Unincorporated nucleotides were removed from the PCR product with the QIAquick PCR purification kit (Qiagen). PCR products were ligated using the TOPO TA cloning kit (Invitrogen) and colony miniprep DNA was prepared for sequencing (GeneWiz, Inc., South Plainfield, NJ). DNAStar Lasergene 8 software was used to analyze sequences.

### Statistical Analyses

The Fisher's exact test was used for statistical comparison of the percentage of SHIV-RT infected animals in the differently treated groups. (GraphPad Prism version 5.02 for Windows, GraphPad Software, San Diego, CA). P values <0.05 were taken as statistically significant.

## Results

### Repeated application of low dose MIV-150 and zinc acetate combination gel prevents vaginal infection by SHIV-RT

**Figure 1:**
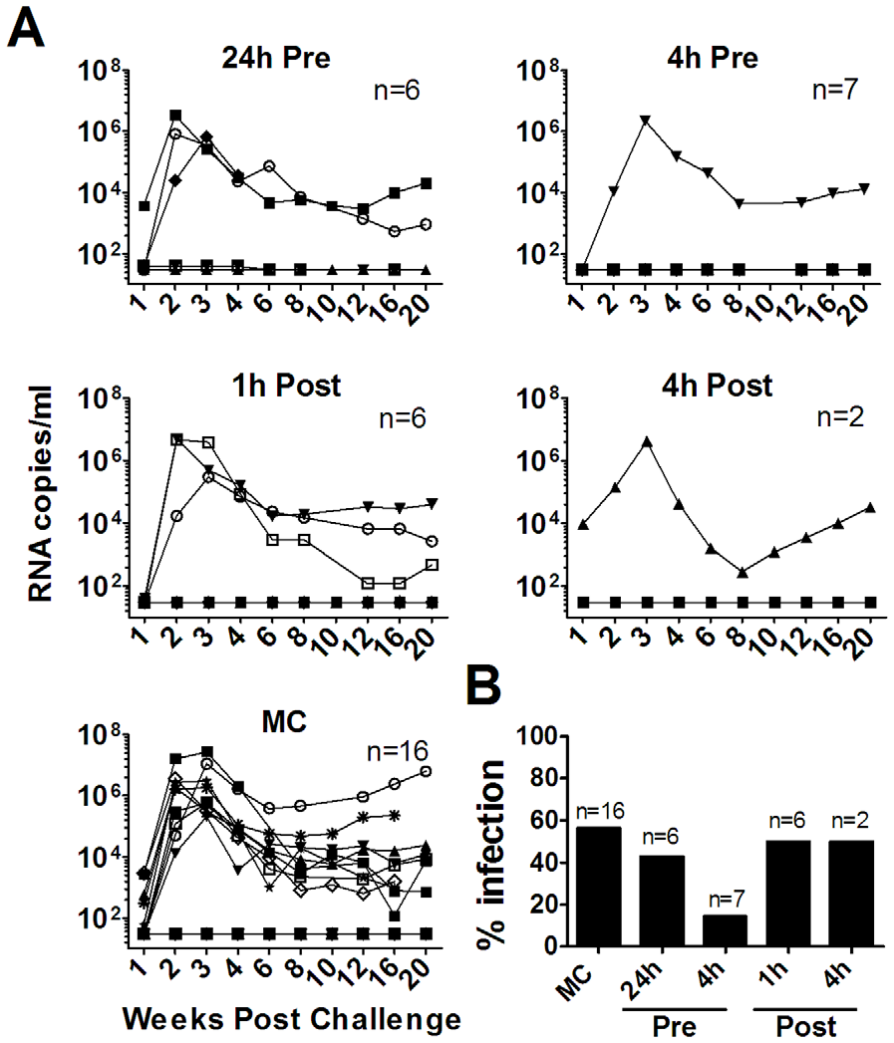
A single dose of 500 µM MIV-150 partially protects against vaginal SHIV-RT infection. **A.** 500 µM MIV-150 in carrageenan (PC-817) or MC placebo were administered 24 or 4 h prior to (Pre) or 1 h or 4 h after (Post) challenge with 10^3^ TCID_50_ SHIV-RT. The number of animals in each treatment group is indicated and animals treated with MC at any time are pooled into one dataset. Plasma viral loads (SIV RNA copies/ml of plasma) were measured over time and each symbol denotes an individual animal. **B.** The percentages of animals infected in each group are summarized.

In previous studies we demonstrated the ability of a 500 µM (185 µg/ml) MIV-150-containing gel to limit vaginal infection when a single dose was applied 30 min prior to SHIV-RT challenge of healthy [20] and HSV-2-infected [22] macaques. In this report we confirmed that there was a trend towards protection for up to 4 h (14% infection compared to 56% infection in the placebo MC-treated animals; p<0.09), but any protection was lost after 24 h, and post-exposure treatment was ineffective (Fig. 1 and Table S1).

**Figure 2:**
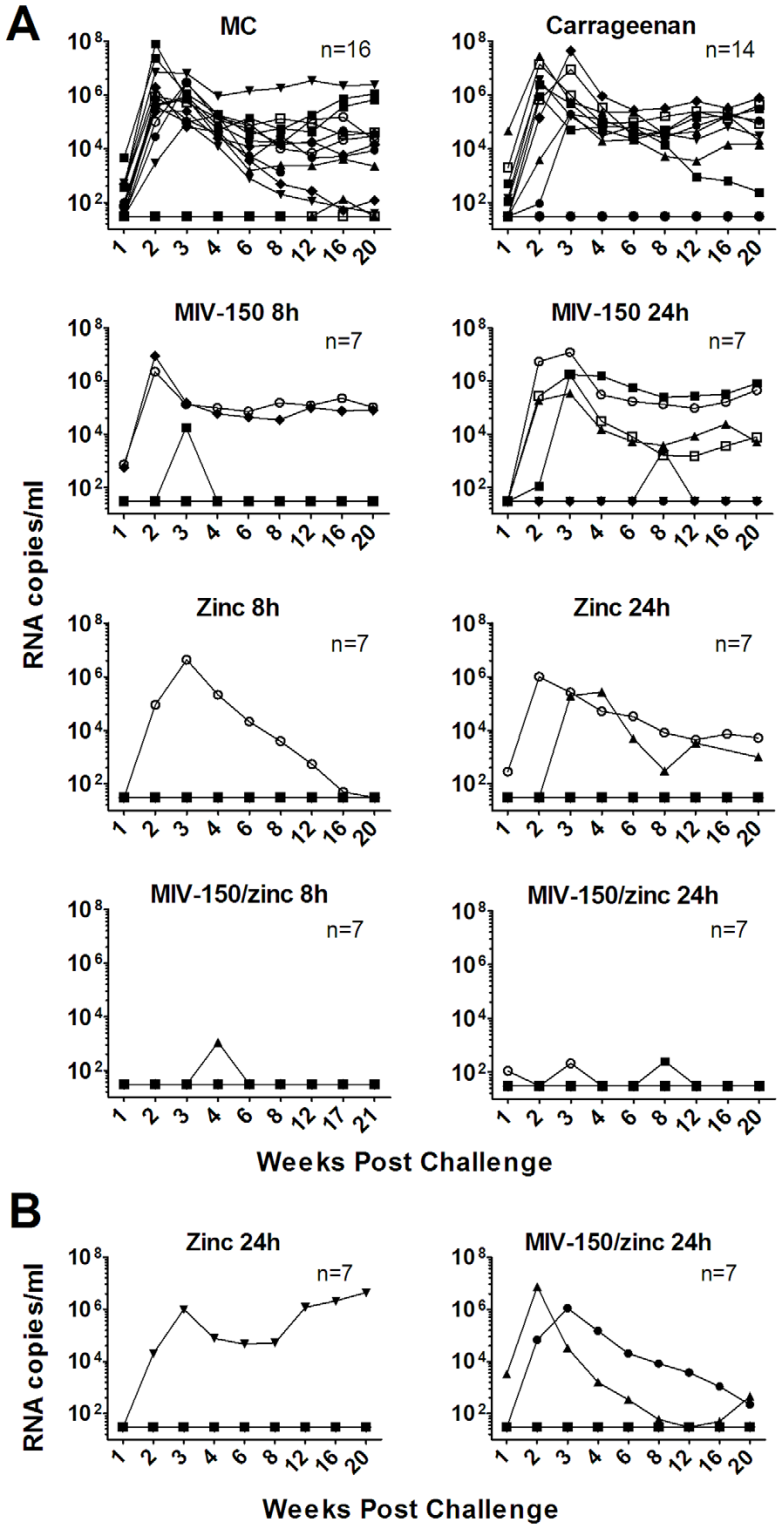
Repeated daily application of 50 µM MIV-150/zinc acetate protects fully for at least 24 h. **A.** Animals were treated daily for 2 weeks with either MC placebo, carrageenan vehicle (PC-515), or carrageenan gel containing 50 µM MIV-150 (PC-815), 14 mM zinc acetate dihydrate (PC-707), or 50 µM MIV-150/14 mM zinc acetate dihydrate (PC-1005). SHIV-RT was then applied vaginally 8 or 24 h after the last gel. The data within the MC and carrageenan controls both represent pooled datasets from different time points (see Table S2). Plasma viral loads were measured over time, and the data for each animal are shown. The numbers of animals in each treatment group are noted. **B.** Animals were treated every other day for 4 weeks with carrageenan gel containing either 14 mM zinc acetate dihydrate or 50 µM MIV-150/14 mM zinc acetate dihydrate, before challenge with SHIV-RT 24 h later. Viral RNA copies/ml are shown.

We then determined if a 10-fold lower dose of MIV-150 (50 µM or 18.5 µg/ml) could be used under a repeated gel-dosing regimen to afford longer lasting protection. Gels were applied daily for 2 weeks, and animals were challenged up to 24 h after the last application. This protocol was intended to model real-world topical gel application by women who would use it on a repeated basis and independently of coitus. Furthermore, this study design would evaluate any detrimental effects of repeated gel exposure. When infected, animals exhibited characteristic viremia with mean peak levels of 2.3×10^6^ RNA copies/ml (typically) at 2–3 weeks post challenge, with mean set point viral loads of 1.7×10^4^ RNA copies/ml being reached by week 8 (across all groups; n = 37). Otherwise, protected animals had no detectable viral RNA for up to 20 weeks of follow-up (<30 copies/ml; n = 56). In six animals, stochastic plasma virus RNA was detected on one or two occasions (blips; confirmed upon repeated testing), but they were otherwise negative for all other time points examined and none of them developed SIV-specific antibody or T cell responses (Tables S2 and S3). Furthermore, no SIV *gag* DNA was detected in the lymph nodes of the six animals with blips (data not shown). Such blips in plasma virus might reflect controlled or aborted infections, as have been reported [39], [40], [41], [42], [43], [44]. Since blips occurred across the different treatment groups, comparisons have been made based on the frequency of animals with typical viremia (not protected) versus those with undetectable virus or virus blips (protected). A MC placebo group was included to control for the non-specific barrier effect of carrageenan that was previously observed when animals were challenged only 30 min after a single gel application [20]. Relative to the MC controls, the barrier effect of the carrageenan vehicle was modest and not statistically significant in this repeated application study, where animals were challenged 8–24 h after the last gel application. Therefore, the protective efficacy has been calculated as the percentage of protection of test gels compared to carrageenan vehicle control (Fig. 2A and Table 1).

**Table 1:** A MIV-150/zinc acetate gel provides significant protection against vaginal SHIV-RT infection.

Gel	Not Protected	Protected	P value vs Carrageenan	Protection vs Carrageenan
**MC**	•••••••••••••	••○	<0.5	
**Carrageenan**	•••••••••	•••••		
**500 µM MIV-150 8 h**	•••	••••	<0.4	33%
**50 µM MIV-150 8 h**	••	••••○	<0.2	56%
**50 µM MIV-150 24 h**	••••	••○	1	11%
**Zinc acetate 8–24 h**	•••	•••••••••••	<0.06	67%
**MIV-150/zinc acetate 4–24 h**		••••••••••••••••••○○○	**<0.0001**	**100%**
**Zinc acetate and MIV-150/zinc acetate, EOD 24 h**	•••	•••••••••••	<0.06	67%

The protected versus not protected animals in the treatment groups shown in Figure 2 and Figures S1 and S2 are summarized. Each symbol marks an individual animal as protected or not. The open symbols denote animals with atypical, low-level viral blips (see text). The data for the animals receiving the daily zinc acetate gels have been pooled since the protection was similar at the two time points (see Table S2). The data for the animals given zinc-containing gels (zinc acetate and MIV-150/zinc acetate) every other day (EOD) have been pooled since similar protection was observed under this regimen (see Figure 2B). The p values (Fisher's exact test) for protection and the percent protection relative to the carrageenan vehicle control group are provided.

Surprisingly, higher doses of MIV-150 offered no protective advantage, with repeated application of the 50 µM MIV-150 gel protecting animals by 56% and 500 µM by 33% relative to vehicle control at the 8 h time point (Fig. 2A and Fig. S1). However, 50 µM MIV-150 afforded no significant protection relative to the vehicle-treated animals and any protective activity of 50 µM MIV-150 was gone after 24 h (Fig. 2A and Table 1).

We next investigated whether adding 14 mM zinc acetate dihydrate, a concentration, which efficiently prevents vaginal and rectal HSV-2 infection in mice (Fernández-Romero, unpublished), would improve protection. Zinc acetate alone provided marked protection against SHIV-RT infection (11 of 14 protected, 67%, p<0.06; Fig. 2A and Table 1). Strikingly, the combination of zinc acetate and 50 µM MIV-150 in carrageenan provided full protection for up to 24 h (p<0.0001; Fig. 2A, Table 1, and Fig. S2). In total, 21 of 21 animals, which received MIV-150/zinc acetate gel, were protected from vaginal SHIV-RT infection.

In order to determine whether daily gel use was required for efficacy, we tested the activity of the two most protective gels, MIV-150/zinc acetate and zinc acetate alone, when applied every other day for 4 weeks, followed by virus challenge 24 h after the last gel. This way, the animals received the same number of total applications as those treated daily for 2 weeks, only over twice the length of time. Both test gels provided protection after every other day administration, but the complete protection seen with the MIV-150/zinc acetate combination after daily treatment was lost (Fig. 2B, Table 1, and Table S3). While we were unable to include the carrageenan vehicle controls for this every other day regimen, comparison to the daily-applied carrageenan control group, in which a greater non-specific barrier effect would be expected than for an every other day dosing regimen, revealed marked protection (67%, 11 of 14 protected; zinc acetate and MIV-150/zinc acetate data combined, p<0.06). Interestingly, when the data from all animals treated with zinc acetate alone were pooled, the protection by zinc acetate was significant (70%, 17 of 21 protected; daily and every other day zinc acetate; p<0.02).

Using a separate group of animals, we also verified that there were no detectable local adverse effects that could explain the differences between the groups of animals repeatedly treated with the zinc and/or MIV-150-containing gels. Vaginal pH and the levels of cytokines and chemokines in fluids were measured before, during and 8 or 24 h after a 2 week administration of the test versus control gels. The presence of zinc acetate or MIV-150 in the formulations had no impact on vaginal pH (Fig. S3A). Low-level CXCL8 and CCL2 responses were detected after exposure to carageenan, although there was no cumulative effect of repeated application (Fig. S3B). While the levels varied between animals (and not all animals responded), inclusion of MIV-150, zinc acetate, or MIV-150 and zinc acetate in the carrageenan formulations had no further impact on the CXCL8 and CCL2 levels (Fig. S3B). Of the factors measured, no other cytokine/chemokines were detected in the vaginal fluids of any of the animals tested over time (not shown).

### Cervix-associated MIV-150 predicts efficacy better than plasma or vaginal tissue MIV-150 levels

**Figure 3:**
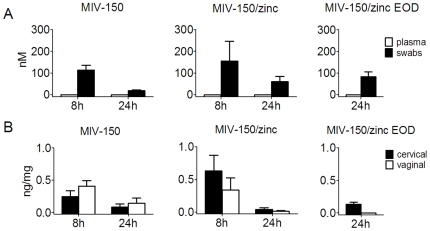
MIV-150 does not accumulate systemically after repeated application. Animals were treated with 50 µM MIV-150 (PC-815) or 50 µM MIV-150/14 mM zinc acetate dihydrate (PC-1005) daily for 2 weeks (daily) or with 50 µM MIV-150/14 mM zinc acetate dihydrate every other day for 4 weeks (EOD). (A) Plasma and vaginal swabs and (B) cervical and vaginal tissues were collected 8 or 24 h after the last gel and the levels of MIV-150 measured by RIA. The mean concentrations (±SEM, n = 6) of MIV-150 for each treatment group are shown.

In a separate set of animals we used RIA to determine the levels of MIV-150 within plasma, vaginal fluids, and vaginal and cervical tissues as a measure of potential systemic absorption and accumulation. Samples were taken after repeated treatment with 50 µM MIV-150 alone (daily) or 50 µM MIV-150 with zinc acetate (daily versus every other day). MIV-150 was undetectable in plasma 8 or 24 h after 2 weeks of daily or 24 h after 4 weeks of every other day treatment with either gel, but it was detected in the vaginal swabs (Fig. 3A). While swab MIV-150 levels were lower 24 h after daily MIV-150 treatment than daily MIV-150/zinc acetate treatment (not statistically significant), comparable levels were detected after every other day dosing with MIV-150/zinc acetate. In order to determine if MIV-150 was being absorbed systemically, followed by rapid clearance, as observed in rats (Rodriguez unpublished), blood samples collected 0.5, 1, 4, 8, and 24 h after daily gel application were tested. MIV-150 remained undetectable at all of these time points after application of either 50 µM MIV-150 alone or MIV-150 and zinc acetate (not shown). Significantly more MIV-150 was present in the swabs (but not blood) after application of the gel containing 500 µM MIV-150 compared to when the 50 µM MIV-150 gel was applied (Fig. S4A). Interestingly, higher amounts of MIV-150 were associated with the cervical tissues 8 h (but not 24 h) after the last (daily) dose of the MIV-150/zinc acetate gel compared to daily dosing of 50 µM MIV-150 alone or every other day dosing of MIV-150/zinc acetate (Fig. 3B). Concentrations associated with the vaginal tissues were comparable 8 h after the last application of the MIV-150/zinc acetate or MIV-150 gels, and even higher in the MIV-150-treated animals at the 24 h time point. In animals treated with 500 µM MIV-150 much more MIV-150 was associated with vaginal tissues and comparable levels were associated with the cervical tissues (compared to the 50 µM MIV-150-treated animals; Fig. S4B).

### Low dose MIV-150-containing gels do not select for infection with drug resistant viruses

Infections occurring in the presence of MIV-150 (observed after daily MIV-150 or every other day MIV-150/zinc acetate treatment) might reflect the selection of drug resistant variants or simply failure of the drug to prevent wild type virus infection. To determine if MIV-150 had selected for drug resistant variants present in the inocula infecting these animals, we sequenced the RT gene of viral RNA isolated from plasma at peak viremia. As in the controls, the RT gene sequences from infected animals that received MIV-150 (with or without zinc acetate) were wild type (Table S4). There were no amino acid changes at positions that confer resistance to NNRTIs [45], [46], [47]. Therefore, infections occurring in the presence of low doses of MIV-150 were not due to the selection of drug resistant variants.

## Discussion

Effective microbicides that can be used frequently and independently of coitus are urgently needed to curb HIV spread worldwide. Although there have been candidate microbicides showing promise in macaques, most required mM or mg/ml amounts of the HIV-targeting anti-viral drugs and delivery was required within minutes or a few hours before the time of virus exposure [1], [2], [3], [4], [5], [6], [7]. Of note, the 1% tenofovir gel that protected macaques when applied just 30 min prior to repeated vaginal challenge [5] was similarly shown to significantly reduce HIV infection in women when used no more than 12 h before and no less than 12 h after intercourse [8]. This is a critical step for the field, providing the first proof of concept that topically applied gels can protect against vaginal HIV infection. Notably, there is increasing interest in the identification of microbicide approaches not based on drugs used as first line therapies against HIV-1 and/or that exhibit cross-resistance, to limit the emergence of drug resistant viruses. We observed absolute protection of macaques against vaginal infection for at least 24 h after daily application of a novel gel comprising a combination of only 50 µM of the NNRTI MIV-150 with 14 mM zinc acetate dihydrate. In contrast, gels containing either component alone afforded only partial protection, with zinc acetate-containing gels providing significant protection when applied daily or every other day (even in the absence of MIV-150). Because of this significant protection, gels with zinc acetate alone should also be considered for further development and human testing, since they are not expected to promote drug resistance.

Macaque models represent an informative system to evaluate microbicide candidates before clinical testing [48]. In fact, vaginal testing of tenofovir gel, using the repeated low dose challenge model, predicted its activity in humans [5], [8]. For greater stringency, we chose to employ a single high dose challenge of animals pretreated with DepoProvera, conditions which increase susceptibility to infection. Our inocula contained 10^3^ TCID_50_ (>1.6×10^6^ RNA copies), which is at least 100-fold greater than the median virus RNA levels typical of human semen [49], [50], [51], [52]. This was combined with applying the gel repeatedly, an administration regimen aimed not only at evaluating protection, but also at identifying any adverse reactions that might augment transmission (since our inoculum was intended not to infect 100% of the control animals). It is interesting to note that there was a trend for increased frequency of infection after repeated application of the placebo MC compared to single MC dosing (81% versus 56%; p<0.2). This was not observed with any of the other gels and, in fact, the infection frequency in the repeated carrageenan vehicle treatment group was comparable to the single treatment MC group (64% versus 56%, p<0.8). In contrast, gels containing zinc acetate or MIV-150/zinc acetate were able to significantly protect against infection in this rigorous test model.

There are only a few reports on the anti-viral activity of zinc salts against HIV and other viruses, including HSV-2 [23], [24], [25]. This is probably largely due to the toxicity often observed *in vitro*, which confounds accurate demonstration of anti-viral activity, and emphasizes the need to evaluate zinc *in vivo*. A more detailed analysis of zinc's mechanism of action is underway to properly understand the basis for the efficacy of our combination gel. We recently found that zinc acetate-containing formulations in carrageenan are extremely effective against vaginal and rectal HSV-2 infection in mice (Fernández-Romero, unpublished), supporting earlier studies reporting zinc's anti-herpetic activity [23], [25]. Therefore, in addition to directly preventing the spread of immunodeficiency viruses as shown here, a zinc-containing gel might block other STIs like HSV-2 and thereby help reduce HIV spread indirectly as well. Zinc has been reported to possess immunomodulatory activities [53], and it is possible that modifications of the cellular milieu within the mucosal tissues render the animals resistant to infection. Our initial studies did not reveal obvious changes in the cytokines and chemokines present in the vaginal fluids, but further *ex vivo* infection and immune studies on biopsies from treated macaques [6] will be useful to dissect the mechanism of zinc acetate's anti-viral activity.

On the other hand, MIV-150 has potent anti-viral properties and may have virucidal activity [13] and, therefore, might act on viruses in the vaginal cavity prior to their entry into the body, as well as within the tissues where infection is established and initially amplified [54]. Pharmacokinetic (PK) studies in rats confirmed that MIV-150 is absorbed after vaginal application, but detection in the blood required 10–100 times more MIV-150 than used herein (Rodriguez, unpublished). These rat studies also showed that MIV-150 has a short plasma half-life, becoming undetectable within 24 h. Therefore, it is not surprising that we did not detect MIV-150 in the plasma after repeated application of gels containing 50 µM MIV-150, but did detect it in the cervico-vaginal fluids and associated with the tissues. This is similar to reports of gels containing the NNRTIs TMC120 or UC-781, where little or no drug was detected in the blood after repeated doses of 0.1–10 mg/ml [55], [56]. The MIV-150 levels in the swabs did not predict efficacy, since (i) similar levels were seen 24 h after the daily versus every other day dosing of MIV-150/zinc acetate, but the protective effect of MIV-150 was not apparent in the latter and (ii) while significantly more MIV-150 was present in the swabs after application of the gel containing 500 µM MIV-150 compared to when the 50 µM MIV-150 gel was applied, the former was not more effective at preventing infection. Rather, the level of MIV-150 associated with the cervical tissues appears to be a better predictor of efficacy. The mean levels of MIV-150 associated with the cervical tissues 8 h after daily treatment with 2 ml of 50 µM MIV-150/14 mM zinc acetate dihydrate (630 pg/mg of tissue) or 50 µM MIV-150 (240 pg/mg) for 2 weeks are higher than the levels of TMC120 reportedly associated with the cervical tissues 8 h after daily treatment for 7 days with 273 µM TMC120 (3–80 pg/mg) [55]. This might be due to differences in the formulations that affect the release of the NNRTI and potentially the association of the drug with the tissues, and/or specific properties of the NNRTIs.

The few animals that became infected in the presence of MIV-150 bore virus expressing the wild type RT gene, indicating that the low doses of MIV-150 used did not select for resistant variants. Viral stocks were grown for a limited number of *in vitro* passages, in the absence of drug selection, and therefore the virus was expected to be clonal at the time of challenge [21], [57]. It is possible that drug resistance will emerge if an infected individual uses an NNRTI-containing gel. However, the favorable resistance profile of MIV-150 and its rapid clearance from the blood, even if it is absorbed (at undetectable levels) after topical application, should lessen the pressure on the virus to select escape variants. Macaque studies are underway to evaluate the emergence of drug resistance during prolonged treatment of infected animals with MIV-150. Although there was no impact of tenofovir on drug resistance within the people who became infected with HIV in the CAPRISA 004 trial [8], tenofovir is used in current HIV therapies. This ultimately increases the chances of transmission of tenofovir resistant viruses (that might develop in HIV-infected people receiving tenofovir therapy), since they would not be blocked by the tenofovir gel. Hence, identifying an effective gel containing novel anti-viral components that are not used in HIV treatment (like MIV-150 and zinc acetate) is critical to also prevent the spread of viruses that become resistant to current treatments.

Unlike earlier studies, we investigated the activity of a gel comprising two distinct active ingredients: the NNRTI MIV-150 and zinc acetate. The prototype formulation of the MIV-150/zinc acetate (and MIV-150) gel tested herein contains 1% DMSO and, while there is no federal regulation or guidance preventing the use of DMSO in microbicides, the promising MIV-150/zinc acetate formulation is currently being optimized without DMSO for human testing. Efficacy at low doses of an NNRTI that is rapidly cleared if absorbed and not already used to treat HIV is highly advantageous as it helps reduce cost, the likelihood of drug resistance, and importantly systemic toxicity. Notably, we demonstrated complete protection by this combination gel after daily usage, an effect that lasted for at least 24 h after the last application. In addition, the significant protective effects of zinc acetate alone, even when used every other day, is encouraging for the development of microbicides that are highly unlikely to select for or induce the development of drug resistant viruses. Safety and (ultimately) efficacy testing of these formulations in humans is required before they can be advanced for human use. These results represent a major step forward for the development of coitally independent microbicide gels that would be used on a frequent basis to help stem the spread of HIV and other STIs.

## Supporting Information

Figure S1 **Limited protection by repeated daily application of 500 µM MIV-150.** Animals (n = 7) were treated daily for 2 weeks with a carrageenan gel containing 500 µM MIV-150 (PC-817). SHIV-RT was then applied vaginally 8 h after the last gel. Plasma viral loads were measured over time, and the data for each animal are shown.
(TIF)

Figure S2 **Repeated daily application of MIV-150/zinc acetate fully protects for 4 h.** Animals (n = 7) were treated daily for 2 weeks with a carrageenan gel containing 50 µM MIV-150 and 14 mM zinc acetate dihydrate (PC-1005). SHIV-RT was then applied vaginally 4 h after the last gel. Plasma viral loads were measured over time, and the data for each animal are shown.
(TIF)

Figure S3 **Repeated application of MIV-150/zinc acetate does not affect vaginal pH or chemokine responses.** Animals were treated with the indicated gels daily for 14 d. Vaginal pH was measured (A) prior to vaginal swabs being collected before gel application (Pre), 1 and 8–9 d during daily gel application (24 h after the previous gel), and 8 and 24 h after the last gel was applied (Post). (B) The CXCL8 and CCL2 levels measured in the vaginal swabs by Luminex are shown. Mean values ± SEM are shown in A and B. Baseline (Pre); n = 23. MC; n = 2 all time points. Carr; n = 6 each time point during treatment, n = 3 each time point post treatment. MIV-150; n = 5 1 d and n = 4 9 d during treatment, n = 1 8 h and n = 3 24 h post treatment. Zinc acetate; n = 5 each time point during treatment, n = 2 8 h and n = 3 24 h post treatment. MIV-150/zinc acetate; n = 5 each time point during treatment, n = 2 8 h and n = 3 24 h post treatment. Samples were taken on day 8 during treatment with MC or zinc acetate, while all others were taken on day 9 during the various treatments.
(TIF)

Figure S4 **MIV-150 levels after daily application of 500 µM MIV-150.** Animals (n = 6) were treated daily for 2 weeks with a carrageenan gel containing 500 µM MIV-150. Blood, vaginal swabs, and vaginal and cervical biopsies were collected 8 h after the last gel was applied. MIV-150 levels were measured by RIA and mean values ± SEM are shown for each.
(TIF)

Table S1 **Infection and immune status of SHIV-RT-challenged macaques after single gel dosing.**
(DOC)

Table S2 **Infection and immune status of SHIV-RT-challenged macaques after daily gel application.**
(DOC)

Table S3 **Infection and immune status of SHIV-RT-challenged macaques after gel application every other day.**
(DOC)

Table S4 **MIV-150 does not select for infection by RT mutant virus.**
(DOC)

## References

[1] Veazey RS, Ketas TA, Klasse PJ, Davison DK, Singletary M (2008). Tropism-independent protection of macaques against vaginal transmission of three SHIVs by the HIV-1 fusion inhibitor T-1249.. Proc Natl Acad Sci U S A.

[2] Veazey RS, Shattock RJ, Pope M, Kirijan JC, Jones J (2003). Prevention of virus transmission to macaque monkeys by a vaginally applied monoclonal antibody to HIV-1 gp120.. Nat Med.

[3] Veazey RS, Klasse PJ, Schader SM, Hu Q, Ketas TJ (2005). Protection of macaques from vaginal SHIV challenge by vaginally delivered inhibitors of virus-cell fusion.. Nature.

[4] Lederman MM, Veazey RS, Offord R, Mosier DE, Dufour J (2004). Prevention of vaginal SHIV transmission in rhesus macaques through inhibition of CCR5.. Science.

[5] Parikh UM, Dobard C, Sharma S, Cong ME, Jia H (2009). Complete protection from repeated vaginal simian-human immunodeficiency virus exposures in macaques by a topical gel containing tenofovir alone or with emtricitabine.. J Virol.

[6] Cranage M, Sharpe S, Herrera C, Cope A, Dennis M (2008). Prevention of SIV rectal transmission and priming of T cell responses in macaques after local pre-exposure application of tenofovir gel.. PLoS Med.

[7] Veazey RS, Ling B, Green LC, Ribka EP, Lifson JD (2009). Topically applied recombinant chemokine analogues fully protect macaques from vaginal simian-human immunodeficiency virus challenge.. J Infect Dis.

[8] Karim QA, Karim SS, Frohlich JA, Grobler AC, Baxter C (2010). Effectiveness and Safety of Tenofovir Gel, an Antiretroviral Microbicide, for the Prevention of HIV Infection in Women.. Science Express Published online July.

[9] Hoare A, Kerr SJ, Ruxrungtham K, Ananworanich J, Law MG (2010). Hidden drug resistant HIV to emerge in the era of universal treatment access in Southeast Asia.. PLos ONE.

[10] Wilkin TJ, Shalev N, Tieu H-V, Hammer SM (2010). Advances in antiretroviral therapy.. Top HIV Med.

[11] Ford N, Calmy A (2010). Improving first-line antiretroviral therapy in resource-limited settings.. Curr Opin HIV AIDS.

[12] Skoler-Karpoff S, Ramjee G, Ahmed K, Altini L, Plagianos MG (2008). Efficacy of Carraguard for prevention of HIV infection in women in South Africa: a randomised, double-blind, placebo-controlled trial.. Lancet.

[13] Fernández-Romero JA, Thorn M, Titchen K, Sudol K, Li J (2007). Carrageenan/MIV-150 (PC-815), A Combination Microbicide.. Sexually Transmitted Diseases.

[14] Kilmarx PH, Blanchard K, Chaikummao S, Friedland BA, Srivirojana N (2008). A randomized, placebo-controlled trial to assess the safety and acceptability of use of carraguard vaginal gel by heterosexual couples in Thailand.. Sex Transm Dis.

[15] Kilmarx PH, van de Wijgert JH, Chaikummao S, Jones HE, Limpakarnjanarat K (2006). Safety and acceptability of the candidate microbicide Carraguard in Thai Women: findings from a Phase II Clinical Trial.. J Acquir Immune Defic Syndr.

[16] Cummins JE, Guarner J, Flowers L, Guenthner PC, Bartlett J (2007). Preclinical testing of candidate topical microbicides for anti-human immunodeficiency virus type 1 activity and tissue toxicity in a human cervical explant culture.. Antimicrob Agents Chemother.

[17] Whitehead SJ, Kilmarx PH, Blanchard K, Manopaiboon C, Chaikummao S (2006). Acceptability of Carraguard vaginal gel use among Thai couples.. AIDS.

[18] Martin S, Blanchard K, Manopaiboon C, Chaikummao S, Scaffer K (2010). Carragaurd acceptability among men and women in a couples study in Thailand.. Journal of Women's Health.

[19] McLean CA, van de Wijgert JH, Jones HE, Karon JM, McNicoll JM (2010). HIV genital shedding and safety of Carraguard use by HIV-infected women: a crossover trial in Thailand.. Aids.

[20] Turville SG, Aravantinou M, Miller T, Kenney J, Teitelbaum A (2008). Efficacy of Carraguard®-based microbicides in vivo despite variable in vitro activity.. PLos ONE.

[21] Uberla K, Stahl-Hennig C, Bottiger D, Matz-Rensing K, Kaup FJ (1995). Animal model for the therapy of acquired immunodeficiency syndrome with reverse transcriptase inhibitors.. Proc Natl Acad Sci U S A.

[22] Crostarosa F, Aravantinou M, Akpogheneta OJ, Jasny E, Shaw A (2009). A macaque model to study vaginal HSV-2/immunodeficiency virus co-infection and the impact of HSV-2 on microbicide efficacy.. PLoS ONE.

[23] Kumel G, Schrader S, Zentgraf H, Daus H, Brendel M (1990). The mechanism of the antiherpetic activity of zinc sulphate.. J Gen Virol.

[24] Haraguchi Y, Sakurai H, Hussain S, Anner BM, Hoshino H (1999). Inhibition of HIV-1 infection by zinc group metal compounds.. Antivir Res.

[25] Arens M, Travis S (2000). Zinc salts inactivate clinical isolates of herpes simplex virus in vitro.. J Clin Microbiol.

[26] Freeman EE, Weiss HA, Glynn JR, Cross PL, Whitworth JA (2006). Herpes simplex virus 2 infection increases HIV acquisition in men and women: systematic review and meta-analysis of longitudinal studies.. Aids.

[27] Kapiga SH, Sam NE, Bang H, Ni Q, Ao TT (2007). The role of herpes simplex virus type 2 and other genital infections in the acquisition of HIV-1 among high-risk women in northern Tanzania.. J Infect Dis.

[28] Abu-Raddad LJ, Magaret AS, Celum C, Wald A, Longini IMJ (2008). Genital herpes has played a more important role than any other sexually transmitted infection in driving HIV prevalence in Africa.. PLos ONE.

[29] Foss AM, Vickerman PT, Heise L, Watts CH (2003). Shifts in condom use following microbicide introduction: should we be concerned?. Aids.

[30] Marx PA, Spira AI, Gettie A, Dailey PJ, Veazey RS (1996). Progesterone implants enhance SIV vaginal transmission and early virus load.. Nat Med.

[31] Maguire RA, Bergman N, Phillips DM (2001). Comparison of microbicides for efficacy in protecting mice against vaginal challenge with herpes simplex virus type 2, cytotoxicity, antibacterial properties, and sperm immobilization.. Sex Transm Dis.

[32] Frank I, Piatak MJ, Stoessel H, Romani N, Bonnyay D (2002). Infectious and whole inactivated simian immunodeficiency viruses interact similarly with primate dendritic cells (DCs): Differential intracellular fate of virions in mature and immature DCs.. J Virol.

[33] Cline AN, Bess JW, Piatak M, Lifson JD (2005). Highly sensitive SIV plasma viral load assay: practical considerations, realistic performance expectations, and application to reverse engineering of vaccines for AIDS.. J Med Primatol.

[34] Frank I, Stoessel H, Gettie A, Turville SG, Bess JW (2008). A fusion inhibitor prevents dendritic cell (DC) spread of immunodeficiency viruses but not DC activation of virus-specific T cells.. J Virol.

[35] Lifson JD, Rossio JL, Piatak M, Parks T, Li L (2001). Role of CD8(+) lymphocytes in control of simian immunodeficiency virus infection and resistance to rechallenge after transient early antiretroviral treatment.. J Virol.

[36] Smith SM, Holland B, Russo C, Dailey PJ, Marx PA (1999). Retrospective analysis of viral load and SIV antibody responses in rhesus macaques infected with pathogenic SIV: predictive value for disease progression.. AIDS Res Hum Retroviruses.

[37] Kumar N, Didolkar AK, Ladd A, Thau R, Monder C (1990). Radioimmunoassay of 7 alpha-methyl-19-nortestosterone and investigation of its pharmacokinetics in animals.. J Steroid Biochem Mol Biol.

[38] Bacci A, Montagnoli C, Perruccio K, Bozza S, Gaziano R (2002). Dendritic cells pulsed with fungal RNA induce protective immunity to Candida albicans in hematopoietic transplantation.. J Immunol.

[39] Ma ZM, Abel K, Rourke T, Wang Y, Miller CJ (2004). A period of transient viremia and occult infection precedes persistent viremia and antiviral immune responses during multiple low-dose intravaginal simian immunodeficiency virus inoculations.. J Virol.

[40] Kim EY, Busch M, Abel K, Fritts L, Bustamante P (2005). Retroviral recombination in vivo: viral replication patterns and genetic structure of simian immunodeficiency virus (SIV) populations in rhesus macaques after simultaneous or sequential intravaginal inoculation with SIVmac239Deltavpx/Deltavpr and SIVmac239Deltanef.. J Virol.

[41] Zhu T, Hu SL, Feng F, Polacino P, Liu H (2004). Persistence of low levels of simian immunodeficiency virus in macaques that were transiently viremic by conventional testing.. Virology.

[42] McChesney MB, Collins JR, Lu D, Lu X, Torten J (1998). Occult systemic infection and persistent simian immunodeficiency virus (SIV)-specific CD4(+)-T-cell proliferative responses in rhesus macaques that were transiently viremic after intravaginal inoculation of SIV.. J Virol.

[43] Miller CJ, Marthas M, Torten J, Alexander NJ, Moore JP (1994). Intravaginal inoculation of rhesus macaques with cell-free simian immunodeficiency virus results in persistent or transient viremia.. J Virol.

[44] Li Q, Estes JD, Schlievert PM, Duan L, Brosnahan AJ (2009). Glycerol monolaurate prevents mucosal SIV transmission.. Nature.

[45] Halvas EK, Wiegand A, Boltz VF, Kearney M, Nissley D (2010). Low frequency nonnucleoside reverse-transcriptase inhibitor-resistant variants contribute to failure of efavirenz-containing regimens in treatment-experienced patients.. J Infect Dis.

[46] Metzner KJ, Giulieri SG, Knoepfel SA, Rauch P, Burgisser P (2009). Minority quasispecies of drug-resistant HIV-1 that lead to early therapy failure in treatment-naive and -adherent patients.. Clin Infect Dis.

[47] Llibre JM, Schapiro JM, Clotet B (2010). Clinical implications of genotypic resistance to the newer antiretroviral drugs in HIV-1-infected patients with virological failure.. Clin Infect Dis.

[48] Veazey RS (2008). Microbicide safety/efficacy studies in animals — macaques and small animal models.. Curr Opin HIV AIDS.

[49] Gupta P, Mellors J, Kingsley L, Riddler S, Singh MK (1997). High viral load in semen of human immunodeficiency virus type 1-infected men at all stages of disease and its reduction by therapy with protease and nonnucleoside reverse transcriptase inhibitors.. J Virol.

[50] Halfon P, Giorgetti C, Khiri H, Penaranda G, Terriou P (2010). Semen may harbor HIV despite effective HAART: another piece in the puzzle.. PLos ONE.

[51] Pasquier C, Saune K, Raymond S, Moinard N, Daudin M (2009). Determining seminal plasma human immunodeficiency virus type 1 load in the context of efficient highly active antiretroviral therapy.. J Clin Microbiol.

[52] Butler DM, Delport W, Kosakovsky Pond SL, Lakdawala MK, Cheng PM (2010). The origins of sexually transmitted HIV among men who have sex with men.. Science Transl Med.

[53] Hirano T, Murakami M, Fukada T, Nishida K, Yamasaki S (2008). Roles of zinc and zinc signaling in immunity: zinc as an intracellular signaling molecule.. Adv Immunol.

[54] Haase AT Targeting early infection to prevent HIV-1 mucosal transmission.. Nature.

[55] Nuttall JP, Thake DC, Lewis MG, Ferkany JW, Romano JW (2008). Concentrations of dapivirine in the rhesus macaque and rabbit following once daily intravaginal administration of a gel formulation of [14C]-dapivirine for 7 days.. Antimicrob Agents Chemother.

[56] Patton DL, Cosgrove Sweeney YT, Balkus JE, Rohan LC, Moncla BJ (2007). Preclinical safety assessments of UC781 anti-human immunodeficiency virus topical microbicide formulations.. Antimicrob Agents Chemother.

[57] Soderberg K, Denekamp L, Nikiforow S, Sautter K, Desrosiers RC (2002). A nucleotide substitution in the tRNAlys primer binding site dramatically increases replication of recombinant simian immunodeficiency virus containing a human immunodeficiency virus type 1 reverse transcriptase.. J Virol.

