# Supplementary figures and images for: An Antiretroviral/Zinc Combination Gel Provides 24 Hours of Complete Protection against Vaginal SHIV Infection in Macaques
Source: PLoS One. 2011 Jan 5;6(1):e15835. doi: 10.1371/journal.pone.0015835 (PMC3016413; doi:10.1371/journal.pone.0015835)

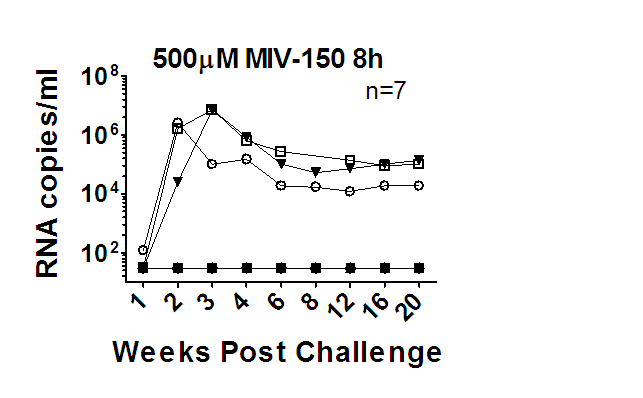

Supplement: Figure S1 — Limited protection by repeated daily application of 500 µM MIV-150. Animals (n = 7) were treated daily for 2 weeks with a carrageenan gel containing 500 µM MIV-150 (PC-817). SHIV-RT was then applied vaginally 8 h after the last gel. Plasma viral loads were measured over time, and the data for each animal are shown. (TIF) [file pone.0015835.s001.tif]

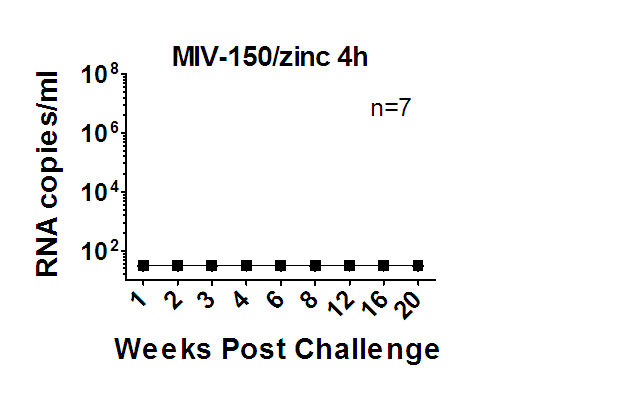

Supplement: Figure S2 — Repeated daily application of MIV-150/zinc acetate fully protects for 4 h. Animals (n = 7) were treated daily for 2 weeks with a carrageenan gel containing 50 µM MIV-150 and 14 mM zinc acetate dihydrate (PC-1005). SHIV-RT was then applied vaginally 4 h after the last gel. Plasma viral loads were measured over time, and the data for each animal are shown. (TIF) [file pone.0015835.s002.tif]

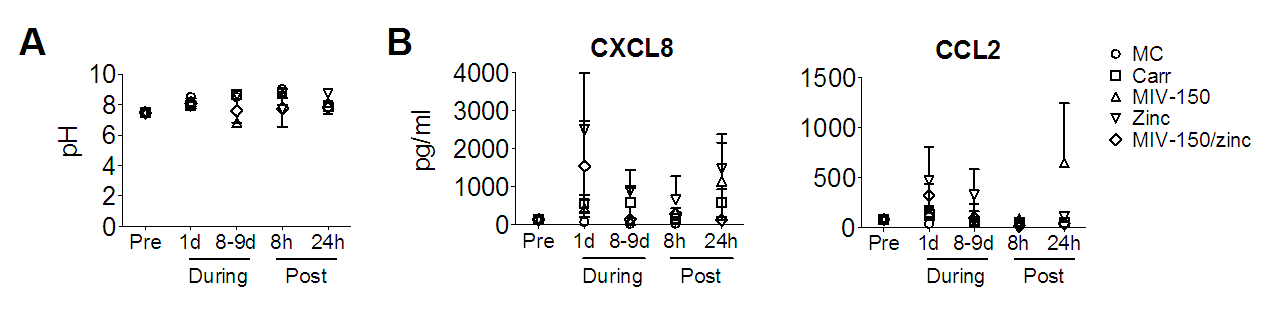

Supplement: Figure S3 — Repeated application of MIV-150/zinc acetate does not affect vaginal pH or chemokine responses. Animals were treated with the indicated gels daily for 14 d. Vaginal pH was measured (A) prior to vaginal swabs being collected before gel application (Pre), 1 and 8–9 d during daily gel application (24 h after the previous gel), and 8 and 24 h after the last gel was applied (Post). (B) The CXCL8 and CCL2 levels measured in the vaginal swabs by Luminex are shown. Mean values ± SEM are shown in A and B. Baseline (Pre); n = 23. MC; n = 2 all time points. Carr; n = 6 each time point during treatment, n = 3 each time point post treatment. MIV-150; n = 5 1 d and n = 4 9 d during treatment, n = 1 8 h and n = 3 24 h post treatment. Zinc acetate; n = 5 each time point during treatment, n = 2 8 h and n = 3 24 h post treatment. MIV-150/zinc acetate; n = 5 each time point during treatment, n = 2 8 h and n = 3 24 h post treatment. Samples were taken on day 8 during treatment with MC or zinc acetate, while all others were taken on day 9 during the various treatments. (TIF) [file pone.0015835.s003.tif]

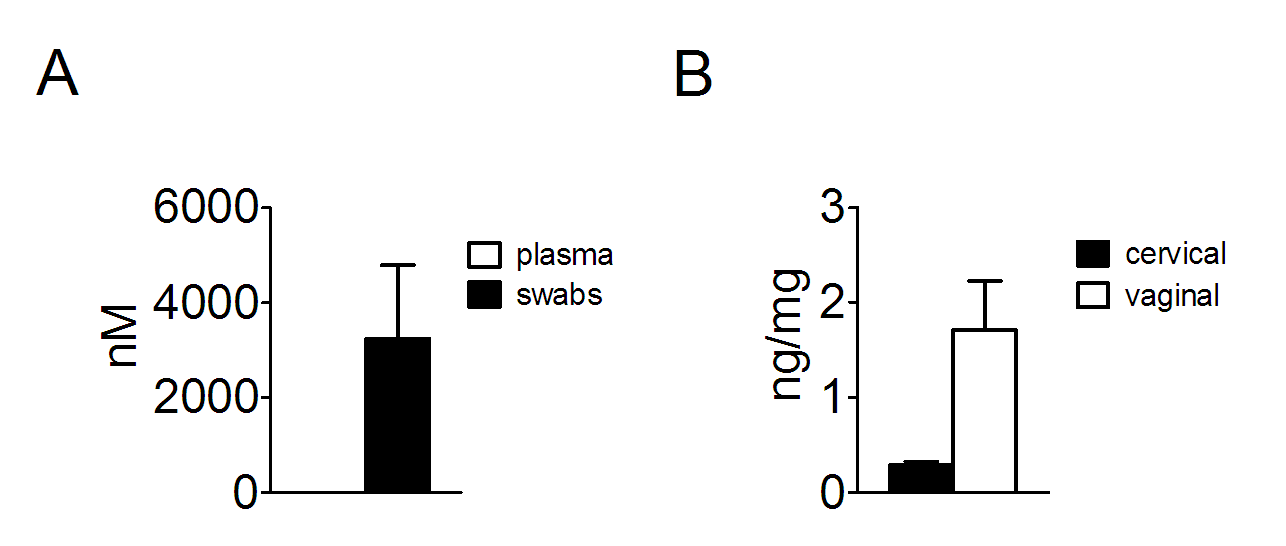

Supplement: Figure S4 — MIV-150 levels after daily application of 500 µM MIV-150. Animals (n = 6) were treated daily for 2 weeks with a carrageenan gel containing 500 µM MIV-150. Blood, vaginal swabs, and vaginal and cervical biopsies were collected 8 h after the last gel was applied. MIV-150 levels were measured by RIA and mean values ± SEM are shown for each. (TIF) [file pone.0015835.s004.tif]
