# Supplementary material for: An Antiretroviral/Zinc Combination Gel Provides 24 Hours of Complete Protection against Vaginal SHIV Infection in Macaques
Source: PLoS One. 2011 Jan 5;6(1):e15835. doi: 10.1371/journal.pone.0015835 (PMC3016413; doi:10.1371/journal.pone.0015835)
Supplement: Table S1 — Infection and immune status of SHIV-RT-challenged macaques after single gel dosing. (DOC) [file pone.0015835.s005.doc]

**Supplementary Table 1. Infection and immune status of SHIV-RT-challenged macaques after single gel dosing.**

| **Gel** | **Challenge time** | **Animal ID^$^** | **Typical viremia** | **Antibody response** | **T cell response** |
| --- | --- | --- | --- | --- | --- |
|
| **MC** |  | FI80 | + | + | + |
|  |  | FI81 | + | + | + |
|  |  | FI82 | + | + | + |
|  |  | DE38^ | - | - | + |
|  |  | GF15 | - | - | - |
|  |  | GF16 | - | - | - |
|  |  | GJ43 | + | + | + |
|  |  | GJ51 | + | + | + |
|  |  | GK06 | - | - | - |
|  |  | GK05 | - | - | - |
|  |  | GF13 | - | - | - |
|  |  | GJ29 | - | - | - |
|  |  | GJ28 | + | + | + |
|  |  | GF13 | + | + | + |
|  |  | HL59 | + | + | + |
|  |  | HL61 | + | + | + |
| **500µM MIV-150** | 24h Pre | GJ70 | + | + | + |
|  |  | GJ84 | - | - | - |
|  |  | GJ33 | + | + | + |
|  |  | GJ37 | - | - | - |
|  |  | GJ79 | - | - | - |
|  |  | GJ84^ | + | + | - |
|  | 4h Pre | HL66 | - | - | - |
|  |  | HL67 | - | - | - |
|  |  | HL68 | + | + | + |
|  |  | HL69 | - | - | - |
|  |  | HL70 | - | - | - |
|  |  | HL71 | - | - | - |
|  |  | HL72 | - | - | - |
|  | 1h Post | GJ34^ | - | + | - |
|  |  | GJ37^ | - | + | - |
|  |  | HL62^ | - | + | - |
|  |  | HL63^ | + | - | - |
|  |  | HL64 | + | + | + |
|  |  | HL65 | + | + | + |
|  | 4h Post | GJ79 | - | - | - |
|  |  | GJ87 | + | + | + |

**Supplementary Table 1 continued**

**^$^** Some animals are listed twice as they were re-enrolled in subsequent studies if they remained uninfected from the initial challenge.

^ SIV-specific B and T cell responses did not correlate with infection status in these animals.
