# Supplementary material for: An Antiretroviral/Zinc Combination Gel Provides 24 Hours of Complete Protection against Vaginal SHIV Infection in Macaques
Source: PLoS One. 2011 Jan 5;6(1):e15835. doi: 10.1371/journal.pone.0015835 (PMC3016413; doi:10.1371/journal.pone.0015835)
Supplement: Table S2 — Infection and immune status of SHIV-RT-challenged macaques after daily gel application. (DOC) [file pone.0015835.s006.doc]

**Supplementary Table 2. Infection and immune status of SHIV-RT-challenged macaques after daily gel application.**

| **Gel** | **Challenge time post gel** | **Animal ID^$^** | **Typical viremia** | **Antibody response** | **T cell response** |
| --- | --- | --- | --- | --- | --- |
|
| **MC** | 4h | HL42 | + | + | + |
|  |  | HL43 | + | + | + |
|  |  | HL45 | + | + | + |
|  |  | HL47 | + | + | + |
|  | 8h | HM17 | + | + | + |
|  |  | HM18 | + | + | + |
|  |  | HM19* | - | - | - |
|  |  | HM20 | + | + | + |
|  |  | GT61 | + | + | + |
|  |  | GT62 | + | + | + |
|  | 24h | HM21 | + | + | + |
|  |  | HM23 | + | + | + |
|  |  | IE88 | + | + | + |
|  |  | IE89 | - | - | - |
|  |  | HL57 | + | + | ND |
|  |  | HL60 | - | - | ND |
| **Carrageenan** | 8h | HM27 | + | + | + |
|  |  | HM28^ | + | + | - |
|  |  | HM29^ | + | + | - |
|  |  | HM30 | + | + | + |
|  |  | HM31 | - | - | - |
|  |  | HM38 | - | - | - |
|  |  | IC82^ | + | + | - |
|  | 24h | IC78 | - | - | - |
|  |  | IC79 | + | + | + |
|  |  | IE78 | + | + | + |
|  |  | IE79 | - | - | - |
|  |  | IE80 | + | + | + |
|  |  | IE81^ | - | + | - |
|  |  | IE82 | + | + | + |
| **500µM MIV-150** | 8h | HM21 | - | - | - |
|  |  | HM22 | - | - | - |
|  |  | HM23 | - | - | - |
|  |  | HM24^ | + | + | - |
|  |  | HM25 | - | - | - |
|  |  | HM26 | + | + | + |
|  |  | IC83 | + | + | + |

**Supplementary Table 2 continued**

| **Gel** | **Challenge time post gel** | **Animal ID^$^** | **Typical viremia** | **Antibody response** | **T cell response** |
| --- | --- | --- | --- | --- | --- |
|
| **50µM MIV-150** | 8h | HM32 | - | - | - |
|  |  | HM33* | - | - | - |
|  |  | HM34 | - | - | - |
|  |  | HM35 | - | - | - |
|  |  | HM36 | + | + | + |
|  |  | HM37 | + | + | + |
|  |  | HM31^ | - | - | + |
|  | 24h | IC80 | - | - | - |
|  |  | IC81 | + | + | + |
|  |  | IE83 | + | + | + |
|  |  | IE84 | - | - | - |
|  |  | IE85* | - | - | - |
|  |  | IE86 | + | + | + |
|  |  | IE87 | + | + | + |
| **14mM zinc acetate** | 8h | HL53 | - | - | - |
|  |  | HL54 | - | - | - |
|  |  | HL55 | - | - | - |
|  |  | HL56 | - | - | - |
|  |  | HL57 | - | - | - |
|  |  | HL58^ | + | + | - |
|  |  | HL60 | - | - | - |
|  | 24h | HL46 | - | - | - |
|  |  | HL48 | - | - | - |
|  |  | HL49^ | + | + | - |
|  |  | HL50 | - | - | - |
|  |  | HL51 | - | - | - |
|  |  | IC84 | - | - | - |
|  |  | GT65^ | + | + | - |
| **MIV-150/zinc acetate** | 4h | HL49 | - | - | - |
|  |  | HL50 | - | - | - |
|  |  | HL51 | - | - | - |
|  |  | HL53 | - | - | - |
|  |  | HL54 | - | - | - |
|  |  | HL60 | - | - | - |
|  |  | GT65 | - | - | - |

**Supplementary Table 2 continued**

| **Gel** | **Challenge time post gel** | **Animal ID^$^** | **Typical viremia** | **Antibody response** | **T cell response** |
| --- | --- | --- | --- | --- | --- |
|
| **MIV-150/zinc acetate** | 8h | HL44 | - | - | - |
|  |  | HL46 | - | - | - |
|  |  | HL48 | - | - | - |
|  |  | HL55 | - | - | - |
|  |  | HL56 | - | - | - |
|  |  | HL57 | - | - | - |
|  |  | HL48* | - | - | - |
|  | 24h | GT57 | - | - | - |
|  |  | GT58* | - | - | - |
|  |  | GT59 | - | - | - |
|  |  | GT60^ | - | - | + |
|  |  | GT64 | - | - | - |
|  |  | GT66* | - | - | - |
|  |  | GT67 | - | - | - |

**^$^** Some animals are listed twice as they were re-enrolled in subsequent studies if they remained uninfected from the initial challenge.

* These animals had random positive plasma virus RNA at one or two time points, but were otherwise below the level of detection.

^ SIV-specific B and T cell responses did not correlate with infection status in these animals. ND is “not determined”.
