# Supplementary material for: An Antiretroviral/Zinc Combination Gel Provides 24 Hours of Complete Protection against Vaginal SHIV Infection in Macaques
Source: PLoS One. 2011 Jan 5;6(1):e15835. doi: 10.1371/journal.pone.0015835 (PMC3016413; doi:10.1371/journal.pone.0015835)
Supplement: Table S3 — Infection and immune status of SHIV-RT-challenged macaques after gel application every other day. (DOC) [file pone.0015835.s007.doc]

**Supplementary Table 3. Infection and immune status of SHIV-RT-challenged macaques after gel application every other day.**

| **Gel** | **Challenge time post gel** | **Animal ID** | **Typical viremia** | **Antibody response** |
| --- | --- | --- | --- | --- |
| **zinc acetate** | 24h | HL55 | - | - |
|  |  | HL48 | - | - |
|  |  | HL46 | - | - |
|  |  | HL50 | + | + |
|  |  | IR25 | - | - |
|  |  | IR26 | - | - |
|  |  | IR27 | - | - |
| **MIV-150/zinc acetate** | 24h | IR29 | + | + |
|  |  | IR30 | - | - |
|  |  | IR31 | + | + |
|  |  | IR32 | - | - |
|  |  | IR33 | - | - |
|  |  | IR34 | - | - |
|  |  | IR35 | - | - |
