# Supplementary material for: An Antiretroviral/Zinc Combination Gel Provides 24 Hours of Complete Protection against Vaginal SHIV Infection in Macaques
Source: PLoS One. 2011 Jan 5;6(1):e15835. doi: 10.1371/journal.pone.0015835 (PMC3016413; doi:10.1371/journal.pone.0015835)
Supplement: Table S4 — MIV-150 does not select for infection by RT mutant virus. (DOC) [file pone.0015835.s008.doc]

**Supplementary Table 4. MIV-150 does not select for infection by RT mutant virus**

| **Gel** | **Animal ID** | **Mutation: L100I, K101P, K103N, V108I, I178L, V179I, Y181C, Y188L, G190E, P225H** |
| --- | --- | --- |
| **MC** | HM18 | 0 (3) |
|  | HL45 | 0 (2) |
|  | HM21 | 0 (9) |
|  | IE88 | 0 (5) |
| **500µM MIV-150** | HM24 | 0 (8) |
|  | HM26 | 0 (4) |
|  | IC83 | 0 (7) |
| **50µM MIV-150** | HM37 | 0 (4) |
|  | HM36 | 0 (8) |
|  | IC81 | 0 (10) |
|  | IE86 | 0 (5) |
|  | IE87 | 0 (6) |
|  | IE83 | 0 (7) |
| **MIV-150/zinc acetate** | IR29 | 0 (11) |
|  | IE31 | 0 (11) |

The number of clones in which amino acid mutations conferring NNRTI resistance were detected is listed for the virus RNA isolated from the indicated animals. The total number of clones sequenced per animal is noted in parentheses.
